# Effect of Xenon Treatment on Gene Expression in Brain Tissue after Traumatic Brain Injury in Rats

**DOI:** 10.3390/brainsci11070889

**Published:** 2021-07-03

**Authors:** Anton D. Filev, Denis N. Silachev, Ivan A. Ryzhkov, Konstantin N. Lapin, Anastasiya S. Babkina, Oleg A. Grebenchikov, Vladimir M. Pisarev

**Affiliations:** 1 Federal Research and Clinical Center of Intensive Care Medicine and Rehabilitology, V. A. Negovsky Research Institute of General Reanimatology, 107031 Moscow, Russia; riamed21@gmail.com (I.A.R.); k.n.lapin@gmail.com (K.N.L.); asbabkina@gmail.com (A.S.B.); oleg.grebenchikov@yandex.ru (O.A.G.); vpisarev@gmail.com (V.M.P.); 2 Research Centre for Medical Genetics (RCMG), 115478 Moscow, Russia; 3 A. N. Belozersky Institute of Physico-Chemical Biology, Moscow State University, Leninskye Gory 1, Building 40, 119992 Moscow, Russia; silachevdn@genebee.msu.su

**Keywords:** TBI, xenon, neuroinflammation, Nanostring, rat, gene expression

## Abstract

The overactivation of inflammatory pathways and/or a deficiency of neuroplasticity may result in the delayed recovery of neural function in traumatic brain injury (TBI). A promising approach to protecting the brain tissue in TBI is xenon (Xe) treatment. However, xenon’s mechanisms of action remain poorly clarified. In this study, the early-onset expression of 91 target genes was investigated in the damaged and in the contralateral brain areas (sensorimotor cortex region) 6 and 24 h after injury in a TBI rat model. The expression of genes involved in inflammation, oxidation, antioxidation, neurogenesis and neuroplasticity, apoptosis, DNA repair, autophagy, and mitophagy was assessed. The animals inhaled a gas mixture containing xenon and oxygen (ϕXe = 70%; ϕO_2_ 25–30% 60 min) 15–30 min after TBI. The data showed that, in the contralateral area, xenon treatment induced the expression of stress genes (*Irf1*, *Hmox1*, *S100A8*, and *S100A9*). In the damaged area, a trend towards lower expression of the inflammatory gene *Irf1* was observed. Thus, our results suggest that xenon exerts a mild stressor effect in healthy brain tissue and has a tendency to decrease the inflammation following damage, which might contribute to reducing the damage and activating the early compensatory processes in the brain post-TBI.

## 1. Introduction

The search for effective methods for neuroprotection and neurorehabilitation after TBI remains a challenge for modern medicine. The use of medical gases represents an approach to overcoming or limiting brain damage post-TBI. Hydrogen, hydrogen sulfide, nitrogen monoxide, carbon dioxide, isoflurane, sevoflurane, oxygen (through hyperbaric oxygenation), and noble gases (helium, argon, and xenon) are currently being studied as neuroprotective gases [1]. Inert or noble gases are considered promising therapeutic molecules, of which xenon is the most extensively researched [2]. A distinctive feature of xenon compared to the other noble gases is its lipophilicity and ability to induce an anesthetic effect at a relatively low pressure (0.4–0.8 atm) [2,3]. Xenon has been employed in clinical medicine for inhalation anesthesia [4]. Several studies have demonstrated cytoprotective effects from xenon in various CNS injuries (trauma; ischemia, including severe neonatal asphyxia; subarachnoid hemorrhage) [5,6,7,8]. For instance, in newborns with severe asphyxia, 72 h-exposure to xenon led to better neurological outcomes [5]. However, little is known about changes in gene expression following xenon treatment after TBI. The potential mechanisms include a decrease in excitotoxicity through blocking NMDA receptors; an improvement in neuronal feeding, increasing the efficiency of metabolite supply; a decrease in inflammation; the alteration of the activity of microglial cells; diminishing apoptosis through BCL2 and TREK-1 proteins [9,10,11,12,13]. Xenon has previously been tested in combination with drugs (ketamine and memantine) for the treatment of neurodegenerative diseases [14,15]. The combined use of one of the drugs with xenon yielded the same results as monotherapy, but at smaller doses, which reduced the side effects of the drug. Some data on xenon’s mechanisms, however, appear to contradict each other. For instance, Breuer T. et al. (2015) noted a proinflammatory effect of xenon (a 3-fold increase in IL-6 and 3-fold decrease in IL-10, compared with sevoflurane) and the induction of oxidative stress in cardiac surgery patients [16]. On the other hand, there are data indicating anti-inflammatory responses to xenon [6]. To identify additional possible mechanisms of xenon treatment after TBI, we studied the dynamics of gene expression for signaling pathways associated with inflammation, antioxidation, autophagy, mitophagy, apoptosis, DNA repair, neurogenesis, and neuroplasticity, as well as the gene expression of potential xenon target proteins (NMDAR, TREK-1, and Clic4) in a TBI model in rats.

## 2. Materials and Methods

### 2.1. Animals

Male Wistar rats (200–300 g) were kept at 3–4 animals per cage, with a 12 h light/dark cycle; water and food were provided ad libitum. The animals (n = 24) were divided into 5 groups, group I (TBI-6 h, n = 5), group II (TBI-6 h + xenon, n = 5), group III (TBI-24 h, n = 5), group IV (TBI-24 h + xenon, n = 5), and group V (intact animals, n = 4). Gene expression analysis was carried out for 3 animals of each group. The animals were randomized into groups according to the subjects’ body weight.

### 2.2. TBI

The rats were anesthetized with an intraperitoneal injection of 300 mg/kg chloral hydrate. Additionally, to ensure effective pain relief in the perioperative and postoperative periods, we used the repeated topical application of a long-acting local anesthetic, bupivacaine ointment. TBI was induced in accordance with the method of dosed contusion injury to the open brain as described previously [17]. The operator performing TBI was blinded to the treatment groups. The head skin in the operating area was shaved and treated with an antiseptic, chlorhexidine 0.05% (ROSBIO, St. Petersburg, Russia). The animal was placed in a stereotaxic frame, the head was fixed, and a skin incision was made along the sagittal suture. Trepanation drilling was performed in the parietal and frontal bones using a 5 mm cutter above the left hemisphere in the sensorimotor cortex area, along with the stereotaxic coordinates 2.5 mm laterally and 1.5 mm caudally, relative to the bregma. The trauma unit was placed so that the striker was above the dura mater. A 50-g mass pin was dropped to the striker from a height of 10 cm. The body temperatures of the rats during surgery were maintained at 37 ± 0.5 °C using a temperature-controlled heating blanket. After the exposure in the anesthesia induction chamber, the animals were warmed up to maintain a rectal body temperature of about 37.0 °C until they woke up (but for no more than 60 min). After waking up (or 1 h later at the end of the exposure), the animals were transferred to a cage.

### 2.3. Xenon Treatment

Within 15–30 min of the TBI, one or two rats were placed in a transparent chamber (5 L) of the Combi-Vet (Rothacher-Medical GmbH, Heitenried, Switzerland) anesthesia machine, and connected to a KNP-01 xenon anesthesia attachment with oxygen and air compressors. A semi-open breathing circuit was used. The passage of the fresh gas mixture through the chamber was ensured by periodic compression of the breathing bag located near the inlet to the chamber. A gas mixture entered the chamber (1 L/min)—achieved concentrations were ϕO_2_ 25–30%/ϕXe 70–75% (KseMed^®^, Akela-N, Khimki, Russia) (groups II and IV) or air (ϕO_2_ 21%) (groups I and III)—for 1 h. The oxygen and xenon concentrations, as well as the gas flow rate, were measured at the chamber inlet using a gas analyzer GKM-03-INSOVT (Insovt, St. Petersburg, Russia) and flowmeters. The animals breathed on their own during the procedure.

### 2.4. Limb-Placing Test

For assessing the neurological status of the animals from groups III and IV (24 h after TBI), a limb-placing test was conducted as described previously [18]. The test was carried out by an experimenter blinded to the treatment groups.

### 2.5. Sampling

The animals were anesthetized with sevoflurane (6 vol.%) and sacrificed. Brain tissue specimens (70–100 mg or 5 × 5 × 2 mm) were harvested from the TBI zone and from the healthy contralateral zone and placed into Eppendorf tubes (2 mL) with RNAlater solution (Thermo Fisher Scientific, Waltham, MA, USA) to stabilize the RNA molecules. According to the manufacturer’s protocol, the samples were kept overnight at +4 °C and then placed in a freezer to store them at −20 °C.

### 2.6. RNA Purification

Total RNA was isolated from brain tissue specimens using the RNeasy Mini kit (Qiagen, Hilden, Germany) and treated with DNase I in accordance with the manufacturers’ protocols. The purity of the isolated RNA was determined spectrophotometrically by using a NanoDrop™ OneC (Thermo Fisher Scientific, Waltham, MA, USA). An RNA Clean & Concentrator—5 kit (Zymo Research, Irvine, CA, USA) was used to remove contaminants from the RNA samples. The concentration of the RNA was assessed using a Qubit 3.0 fluorometer (Thermo Fisher Scientific, Waltham, MA, USA). The RNA integrity number (RIN) was determined electrophoretically on an Agilent 2100 bioanalyzer (Agilent Technologies, Santa Clara, CA, USA) using an Agilent RNA 6000 Nano Kit (Agilent Technologies, Santa Clara, CA, USA); only samples with RIN > 5 were used for further analysis.

### 2.7. Multiplex Gene Expression Analysis

To explore the expression of 91 genes (for the full list of target genes, see Table S1) in the brain tissue specimens, the nCounter FLEX Analysis System and the CodeSet reagent kit (Nanostring Technology, Inc., Seattle, WA, USA) were employed. This technology allows the direct assay of gene transcriptional activity in a multiplex manner. The method is based on the labeling of targets with unique color barcodes attached to target-specific probes and their subsequent detection. Analysis was performed according to the manufacturer’s protocol.

### 2.8. Statistics

Analysis of variance (One-way ANOVA, the Holm–Sidak method) was performed using SigmaStat 3.1 (Systat Software Inc., San Jose, CA, USA). Two-fold differences in expression vs. control were considered significant at *p* < 0.00055 and a tendency at 0.00055 < *p* < 0.05 for target genes, and were considered significant at *p* < 0.05 for HKGs. The control values for housekeeping genes were calculated as described in the Results (3.1). The variability was assessed using the web page https://ncalculators.com/statistics/coefficient-of-variance-calculator.htm (accessed on April–May 2021).

## 3. Results

### 3.1. Housekeeping Gene Selection

There are various approaches to the assessment of housekeeping genes (HKGs) [19]. However, what the optimal method is remains controversial. Based on literature screening, we selected 5 HKGs, *B2m, Gapdh, Hmbs, Ppia*, and *Ywhaz*, employed in various studies as proven HKGs [20,21,22]. We did not find any data on the optimal HKG for TBI in rats receiving xenon treatment. Therefore, the selected HKGs were analyzed using the coefficient of variation (CV) and comparing the means of the changes within the groups.

**Figure 1:**
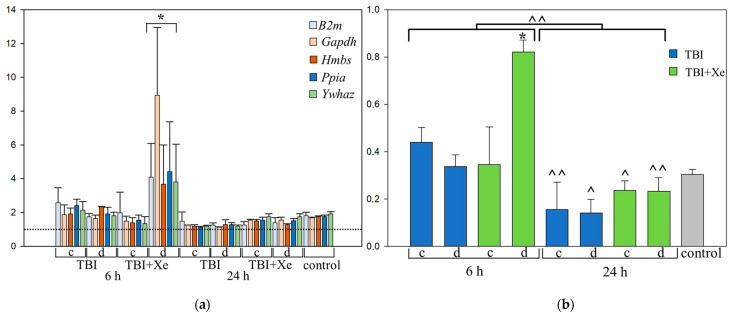
Housekeeping gene variations. (**a**) Mean of changes in the expression of HKGs in each group. (**b**) Coefficient of variation of HKG expression in brain tissue after TBI. * *p* < 0.05 to each group; 24 h vs. 6 h: ^ *p* < 0.05, ^^ *p* < 0.0001. One-way ANOVA, Holm–Sidak method; c—contralateral area, d—damaged area. www.BioRender.com.

The analysis revealed that the CV of *B2m* gene expression was the highest (40%) (Table 1). We analyzed the frequencies of changes in the expression of the HKGs in each group. The greatest changes (at least two-fold, *p* < 0.00001) were observed in the 6h-TBI-xenon group in the injury zone (Figure 1b), while the *Gapdh* gene showed the greatest variation (*p* > 0.05) (Figure 1a).

We analyzed whether there were changes in the CVs of the HKGs in the TBI rats compared to those in the intact animals. After 6 h, there was a 1.28-fold increase in variability (*p* = 0.0555); after 24 h, there was a more than 2-fold decrease (*p* = 0.00124). The animals who inhaled xenon after TBI showed a more pronounced increase in the CVs of the HKGs than the intact animals at 6 h (1.93-fold, *p* = 0.0092), while the CVs were close to those for the intact animals at 24 h after the injury (*p* = 0.497).

Thus, xenon significantly increased the variability of all 5 HKGs in the damaged area at 6 h, especially *Gapdh*. Based on the data obtained on the variability of the HKGs’ expression, three HKGs were chosen: *Hmbs*, *Ppia*, and *Ywhaz*. Gene expression of the target genes was normalized to the chosen HKGs in each experimental group and time-point.

### 3.2. Xenon and Targeted Genes

The most notable changes in gene expression after xenon treatment were found in 4 of the 91 target genes: the proinflammatory *Irf1* gene; the gene of the main antioxidant enzyme in the brain, *Hmox1*; genes for the S100 family proteins associated with neuroinflammation, neuroprotection and neuritogenesis—*S100A8* and *S100A9* [23,24,25].

**Figure 2:**
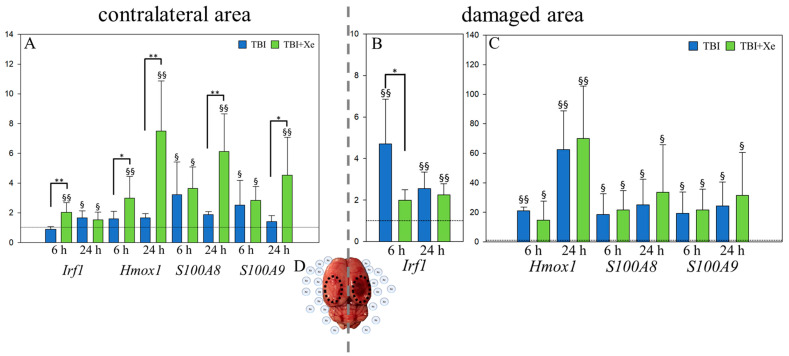
Gene expression analysis. (**A**) Gene expression in the contralateral region. (**B**) *Irf1* gene expression in the damaged area. (**C**) *Hmox1, S100A8*, and *S100A9* gene expression in the damaged area. TBI vs. TBI + Xe: * *p* < 0.05; ** *p* < 0.00055. ^§^ *p* < 0.05 and ^§§^ *p* < 0.00055, compared to intact animals. (**D**) The state of the rat brain 6 h after TBI with selected contralateral (**left**) and damaged (**right**) areas. One-way ANOVA, Holm-Sidak method.

**Figure 3:**
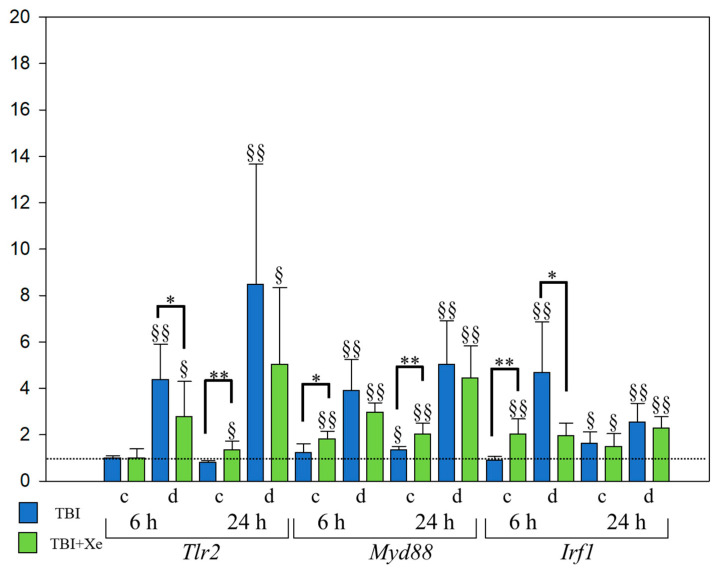
Inflammatory gene expression in the rat brain tissue with TBI. TBI vs. TBI + Xe: * *p* < 0.05, ** *p* < 0.00055; ^§^ *p* < 0.05, ^§§^ *p* < 0.00055, compared to intact animals. One-way ANOVA, Holm-Sidak method.

In the contralateral area, in the TBI + Xe group vs. the TBI group, the expression of *Irf1* was increased 2.3-fold at 6 h (*p* < 0.00055), while *Hmox1* was increased 4.5-fold (*p* < 0.00055) and *S100A8* 3.3-fold (*p* < 0.00055) at 24 h (Figure 2a). The expression of *Tlr2* was increased 1.8-fold (*p* < 0.00055) and *Myd88* 1.6-fold (Figure 3) (*p* < 0.00055) at 24 h. There was a tendency toward increased expression of the *Hmox1* gene (1.9-fold) at 6 h, and that of the *S100A9* gene was increased 3.3-fold at 24 h after TBI.

The *Irf1* gene expression of the TBI + Xe group was 2.4-fold lower than that of the TBI group in the damaged area (*p* = 0.0021) (Figure 2B,C and Figure 3).

### 3.3. Limb-Placing Test

**Figure 4:**
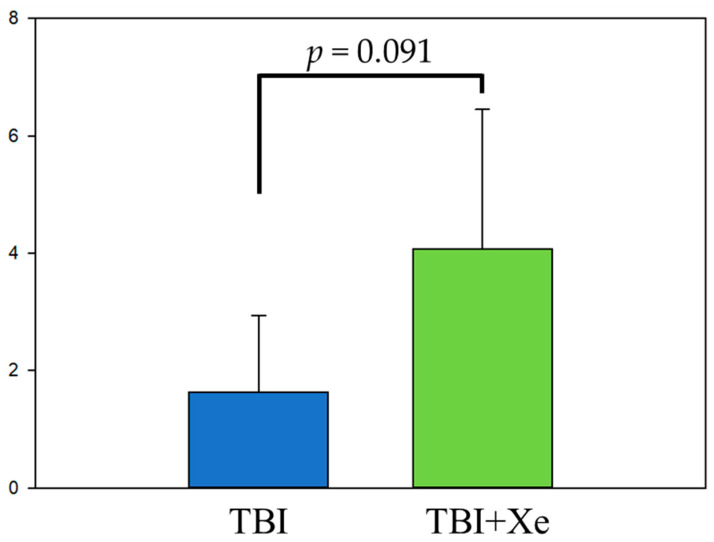
Limb-placing test in 24 h after TBI. Ordinate—the number of test scores.

The neurological status was assessed in the rats from the TBI group (n = 5) and the TBI + xenon group (n = 5) at 24 h after TBI by the limb-placing test. The animals that inhaled xenon exhibited 2.5-fold-higher test scores than the TBI group (*p* = 0.091) (Figure 4), indicating a trend toward less motor weakness due to xenon.

## 4. Discussion

Noble gases, due to their chemical inertness, are attractive targets for use as neuroprotectants. Various noble gases are being tested, but xenon is the most common for clinical studies [2]. Despite many data indicating xenon’s clinical efficacy [5,6,7], the effect of xenon on the expression of genes involved in neuroprotection and their transient expression after TBI remain poorly understood. In this study, we observed a high variability in the expression of HKGs, within the damaged area in the 6h-TBI-Xe group, which could be a potential confounder. Three HKGs with less-variable expression (*Ppia*, *Ywhaz*, and *Hmbs*) were chosen as internal controls for the gene expression study. An additional search for the most stable HKGs at different time points may be required to prove the effects of xenon in a TBI rat model.

An explanation of these possible effects of xenon, is its ability to stabilize the membranes of brain cells [26], binding at the glycine site of the NMDA receptor (GluN1 subunit) [10,27]. When xenon is removed, a “withdrawal syndrome” occurs, triggering lipid peroxidation (LPO), with an adequate response by brain cells observed [26]. The xenon therapy regimen used in newborns with severe asphyxia by Amer A.R. (2018) appears to be optimal in terms of the anti-inflammatory effect and protection against LPO afterward: it entails long-term xenon treatment (up to 72 h) in the asphyxia acute period in combination with therapeutic hypothermia [5]. A gradual increase in a baby’s body temperature for 4 h or more after the procedure [28] will probably contribute to a slow increase in or a blockade of LPO when xenon is eliminated. However, there are no data on the consequences of mild post-xenon LPO. Apparently, in response to xenon, both inflammatory and antioxidative genes are activated in intact brain tissue, which was observed in the current study: the expression of an inflammatory gene (*Irf1*) the main gene of the antioxidant system (*Hmox1*), and genes associated with neurogenesis and neuroplasticity (*S100A8* and *S100A9*) increased. Notably, *Hmox1*’s expression was elevated for longer and more strongly, for up to 24 h, than *Irf1*’s. Early increases in the contralateral area expression of the antioxidant *Hmox1* gene and *S100A8/S100A9* genes encoding the dimer calgranulin contribute to neuroplasticity [25], and might protect normal neurons from secondary damage (pro-oxidant stress signaling products from the damaged area including circulating DNA and other DAMP molecules). That xenon-induced feature in normal brain tissue might be crucial for developing both early and long-term neuroprotection post-TBI. Indeed, xenon-induced long-term (20 months) protection was associated with reduced white matter loss and neuronal loss in the corpus callosum and hippocampal CA1 and dentate in a contralateral area in mice post-TBI [6]. Whether this protective effect of xenon is true for mice of different ages is still unknown, since aging may diminish xenon’s cytoprotection because of alterations of microcirculation in aging brain tissue causing less accumulation of the gas molecules [29].

There was a decrease (tendency) in the expression of the inflammatory genes *Irf1* and *Tlr2* at 6 h in the damaged area. This might be associated with the stabilization of the cytoplasmic and organoid membranes of activated immune cells of the nervous tissue and blood by xenon and its demonstrated anti-inflammatory effect.

Lastly, the rats’ neurological status 24 h after the injury and xenon treatment was evaluated. Xenon treatment (postconditioning) resulted in a better neurological outcome (tendency), as observed by Campos-Pires R. (2020) in a rat TBI model [8].

The study has some limitations. The animals from TBI and TBI + Xe received different concentrations of oxygen in the induction chamber (Δ ϕO_2_ = 5–9%). A high concentration of inhaling oxygen in rats leads to the alteration of inflammatory and antioxidative genes [30,31,32]. However, the concentration of oxygen is much higher (ϕO_2_ more than 80%) than in our study. Therefore, the difference in O_2_ in the inhaling air between the TBI and TBI + Xe groups was neglected.

Thus, xenon treatment or postconditioning in TBI has the potential to reduce inflammation in the brain-damaged area, and at the same time, it represents a mild stressor agent for intact brain tissue, which probably contributes to the activation of compensatory mechanisms in the brain, improving neurological outcomes.

## Figures and Tables

**Table 1:** Expression stability of candidate reference genes in sensorimotor region in TBI rats treated with xenon, evaluated using coefficient of variation (CV).

HKG	CV%	SD	Rank
*Gapdh*	28.6	20.2	1
*Ppia*	28.8	22.1	2
*Ywhaz*	29.8	18.5	3
*Hmbs*	30.1	18.2	4
*B2m*	40.0	19.1	5

## Supplementary Materials

The following are available online at https://www.mdpi.com/article/10.3390/brainsci11070889/s1, Table S1: The panel of genes for multiplex gene expression analysis.
