# Supplementary material for: Effect of Xenon Treatment on Gene Expression in Brain Tissue after Traumatic Brain Injury in Rats
Source: Brain Sci. 2021 Jul 3;11(7):889. doi: 10.3390/brainsci11070889 (PMC8301933; doi:10.3390/brainsci11070889)
Supplement: Supplementary file 1 [file brainsci-11-00889-s001.zip › brainsci-1248119-supplementary.pdf]

Supplementary materials

Table S1. The panel of genes for multiplex gene expression analysis

Inflammation			Apoptosis	DNA repair	Neurogenesis and neuroplasticity	Oxidation and antioxidation	mTOR signalling	Autophagy and mitophagy	Others	HKGs
S100b	*Caspase-1*	*Nfkb1*	*Bax*	*Ogg1*	*TrkB*	*Sod1*	*Prkaa2*	*Pink1*	*Kcnk2*	*Ppia*
S100A8	*Caspase-11*	*Nfkb2*	*Bcl2*	*Parp1*	*Mapk1*	*Nrf2*	*Akt1*	*Atg5*	*Nmdar*	*Hmbs*
S100A9	*Tlr2*	*Myd88*	*Survivin*	*H2AX*	*Igf-1*	*Hmox1*	*Mtor*	*Csnk2a2*	*Daxx*	*B2m*
Il1b	*Tlr4*	*Cmpk2*	*Tgfb*	*Brca1*	*Ngfr*	*Nqo1*	*Akt1s1*	*Mfn1*	*Aqp4*	*Gapdh*
Il6	*Tlr9*	*Nlrp3*	*Smad4*		*Plcg1*	*Nox4*	*Akt2*	*Sqstm1*	*Aqp5*	*Ywhaz*
Il10	*Irf1*	*Aim2*	*Bad*		*Creb*	*Nox2*		*Src*	*Clic4*	
Il18	*Irf3*	*Nod1*			*Bdnf*	*Hif1a*		*Ulk1*	*Gsk3b*	
CD14	*Tnfa*	*Nod2*			*Pi3k*	*Keap1*		*Beclin-1*	*b-Catenin*	
CD36	*Tmem173 (Sting1)*	*Mb21d1 (cGAS)*			*Jnk*	*Gclc*		*Map1lc3a*		
Olr1	*Hmgb1*	*Cxcl1*			*Vegf*	*Park7*		*Mfn2*		
Wnt3a	*Gsdmd*	*Icam1*			*Ncam1*					
Wnt5a					*Gdnf*					
					TrkA					
					Ngf					
